# The influence of bright and dim light on substrate metabolism, energy expenditure and thermoregulation in insulin-resistant individuals depends on time of day

**DOI:** 10.1007/s00125-021-05643-9

**Published:** 2022-02-02

**Authors:** Jan-Frieder Harmsen, Jakob Wefers, Daniel Doligkeit, Luc Schlangen, Bas Dautzenberg, Pascal Rense, Dirk van Moorsel, Joris Hoeks, Esther Moonen-Kornips, Marijke C. M. Gordijn, Wouter D. van Marken Lichtenbelt, Patrick Schrauwen

**Affiliations:** 1 Department of Nutrition and Movement Sciences, NUTRIM School for Nutrition and Translational Research in Metabolism, Maastricht University Medical Center, Maastricht, the Netherlands; 2 Human-Technology Interaction Group and Intelligent Lighting Institute, Department of Industrial Engineering and Innovation Sciences, Eindhoven University of Technology, Eindhoven, the Netherlands; 3 Division of Endocrinology, Department of Internal Medicine, Maastricht University Medical Center, Maastricht, the Netherlands; 4 Chronobiology Unit, Groningen Institute for Evolutionary Life Sciences, University of Groningen, Groningen, the Netherlands; 5 Chrono@Work, Groningen, the Netherlands

**Keywords:** Biological clock, Circadian rhythm, Glucose intolerance, Insulin resistance, Light at night, Light exposure, Melatonin, Postprandial metabolism, Sleeping metabolic rate

## Abstract

**Aims/hypothesis:**

In our modern society, artificial light is available around the clock and most people expose themselves to electrical light and light-emissive screens during the dark period of the natural light/dark cycle. Such suboptimal lighting conditions have been associated with adverse metabolic effects, and redesigning indoor lighting conditions to mimic the natural light/dark cycle more closely holds promise to improve metabolic health. Our objective was to compare metabolic responses to lighting conditions that resemble the natural light/dark cycle in contrast to suboptimal lighting in individuals at risk of developing metabolic diseases.

**Methods:**

Therefore, we here performed a non-blinded, randomised, controlled, crossover trial in which overweight insulin-resistant volunteers (*n* = 14) were exposed to two 40 h laboratory sessions with different 24 h lighting protocols while staying in a metabolic chamber under real-life conditions. In the Bright day–Dim evening condition, volunteers were exposed to electric bright light (~1250 lx) during the daytime (08:00–18:00 h) and to dim light (~5 lx) during the evening (18:00–23:00 h). Vice versa, in the Dim day–Bright evening condition, volunteers were exposed to dim light during the daytime and bright light during the evening. Randomisation and allocation to light conditions were carried out by sequential numbering. During both lighting protocols, we performed 24 h indirect calorimetry, and continuous core body and skin temperature measurements, and took frequent blood samples. The primary outcome was plasma glucose focusing on the pre- and postprandial periods of the intervention.

**Results:**

Spending the day in bright light resulted in a greater increase in postprandial triacylglycerol levels following breakfast, but lower glucose levels preceding the dinner meal at 18:00 h, compared with dim light (5.0 ± 0.2 vs 5.2 ± 0.2 mmol/l, *n* = 13, *p*=0.02). Dim day–Bright evening reduced the increase in postprandial glucose after dinner compared with Bright day–Dim evening (incremental AUC: 307 ± 55 vs 394 ± 66 mmol/l × min, *n* = 13, *p*=0.009). After the Bright day–Dim evening condition the sleeping metabolic rate was identical compared with the baseline night, whereas it dropped after Dim day–Bright evening. Melatonin secretion in the evening was strongly suppressed for Dim day–Bright evening but not for Bright day–Dim evening. Distal skin temperature for Bright day–Dim evening was lower at 18:00 h (28.8 ± 0.3°C vs 29.9 ± 0.4°C, *n* = 13, *p*=0.039) and higher at 23:00 h compared with Dim day–Bright evening (30.1 ± 0.3°C vs 28.8 ± 0.3°C, *n* = 13, *p*=0.006). Fasting and postprandial plasma insulin levels and the respiratory exchange ratio were not different between the two lighting protocols at any time.

**Conclusions/interpretation:**

Together, these findings suggest that the indoor light environment modulates postprandial substrate handling, energy expenditure and thermoregulation of insulin-resistant volunteers in a time-of-day-dependent manner.

**Trial registration:**

ClinicalTrials.gov NCT03829982.

**Funding:**

We acknowledge the financial support from the Netherlands Cardiovascular Research Initiative: an initiative with support from the Dutch Heart Foundation (CVON2014–02 ENERGISE).

**Graphical abstract:**

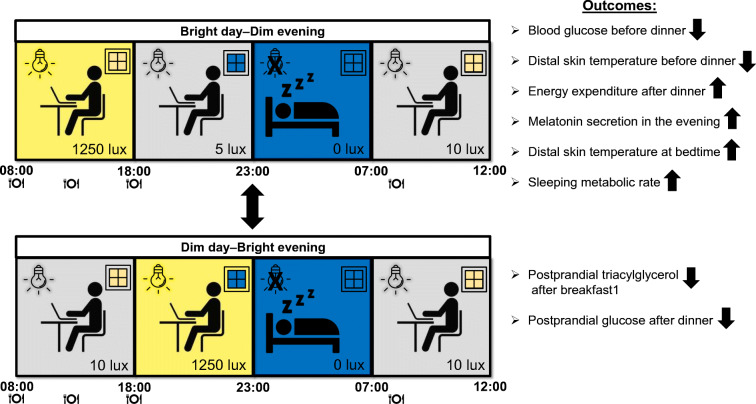

**Supplementary Information:**

The online version contains peer-reviewed but unedited supplementary material available at 10.1007/s00125-021-05643-9.

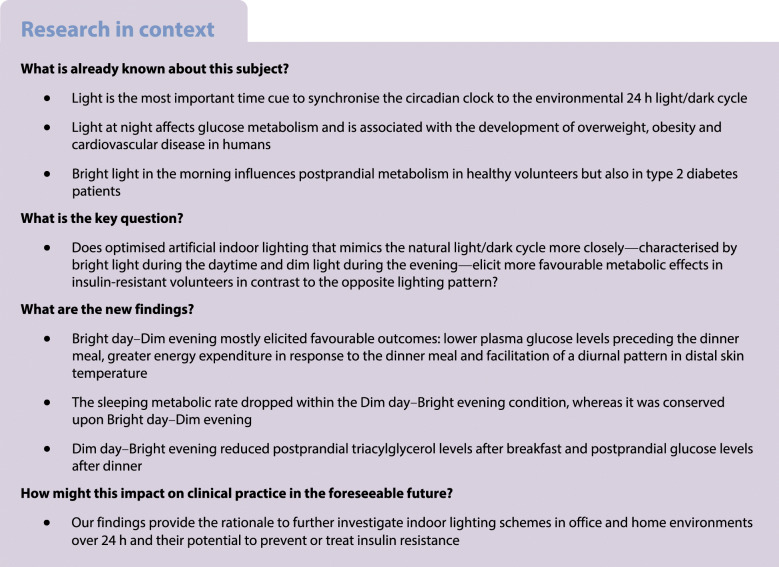



## Introduction

Light is the most important time cue, i.e. zeitgeber, to synchronise the inner biological clock of mammals to the environmental 24 h light/dark cycle. The suprachiasmatic nucleus located in the hypothalamus acts as a master pacemaker by integrating the light/dark information received from the retina with endogenously generated rhythms into neural and humoral signals, which in turn can synchronise peripheral clocks in organs such as the liver, skeletal muscle and pancreas [1–3]. In this manner, the organism can anticipate the different metabolic demands associated with a particular time slot within the light/dark cycle, such as feeding and fasting, and physical activity and rest. Optimising indoor light conditions to more closely mimic the natural light/dark cycle likely ensures proper regulation of behavioural rhythms, including the sleep/wake and feeding cycles, and helps in maintaining metabolic health.

However, in our modern society, artificial light is available around the clock and most people expose themselves to electrical light and light-emissive screens during the dark period of the natural light/dark cycle. In humans, the detrimental effects of light at night (LAN) include acutely elevated postprandial glucose and insulin levels [4] and elevated postprandial insulin and glucagon-like peptide-1 levels after breakfast following light exposure during the night [5]. In a prospective cohort study, Obayashi et al [6] found that LAN exposure increases the incidence of type 2 diabetes mellitus in the elderly population. In addition to LAN, our modern society also faces a lack of sufficient time spent under bright light conditions during the daytime [7, 8] and, in fact, most time is spent indoors under artificial lighting (under much lower light levels compared with natural daylight outdoors). Bright light exposure before and during breakfast increased postprandial glucose and triacylglycerol (TG) levels in type 2 diabetes mellitus patients, whereas it only increased TG levels in young healthy men [9]. A few studies investigated experimental light exposure protocols over 24 h and suggested that bright light can influence glucose metabolism in healthy young volunteers at different times of the day when compared with dim light [10, 11]. However, whether optimising artificial light exposure over a 24 h period can improve whole-body energy and substrate metabolism and glucose homeostasis in individuals with insulin resistance has not yet been investigated.

Here, we investigated the hypothesis that a combination of bright light during the daytime and dim light during the evening would elicit favourable metabolic effects compared with a combination of dim light during the daytime and bright light during the evening. To this end, we performed 24 h metabolic phenotyping by means of indirect calorimetry and frequent blood sampling for TG, glucose, insulin and melatonin concentrations upon two different lighting protocols in a randomised crossover design in insulin-resistant volunteers.

## Methods

The study consisted of a randomised crossover protocol with two arms. The Medical Research Ethics Committee of Maastricht University Medical Center approved the study protocol. All participants provided written informed consent before enrolment in the experiment. All procedures were conducted in accordance with the Declaration of Helsinki. Experiments took place between July 2018 and November 2019. The study was registered at ClinicalTrials.gov (registration no. NCT03829982).

### Participants

Male and female, overweight, insulin-resistant volunteers (40–75 years) were recruited. Insulin resistance was defined as having at least one of the following: (1) impaired fasting glucose: 6.1–6.9 mmol/l; (2) impaired glucose tolerance: 7.8–11.1 mmol/l 2 h after consumption of a 75 g glucose drink; (3) HbA_1c_ of 39–46 mmol/mol (5.7–6.4%); or (4) low insulin sensitivity defined as a glucose clearance rate ≤360 ml kg^−1^ min^−1^ according to the Oral Glucose Insulin Sensitivity model [12]. Participants were non-smokers and generally healthy. Only participants with a habitual bedtime of 23:00 h ± 2 h and 7–9 h of sleep per day were included, and participants were excluded if they performed shift work or had travelled across more than one time zone in the 3 months before the study. Additional exclusion criteria were (history of) cardiovascular diseases and regular medication use that could interfere with study outcomes. Using the Morningness–Eveningness Questionnaire Self-Assessment Version 1.3 (MEQ-SA; score: 35–70), extreme early or late chronotypes were excluded. Baseline participant characteristics are shown in Table 1.

**Table 1:** Baseline participant characteristics

Variable	Mean ± SD
Age (years)	67 ± 6
Sex (female/male)	4/10
Height (m)	1.72 ± 0.06
Body weight (kg)	88 ± 12
BMI (kg/m^2^)	29.6 ± 3.0
Body fat (%)	38.4 ± 6.2
Habitual sleep duration (h)	7.9 ± 0.6
Habitual bedtime (h)	23:34 ± 00:45
MEQ-SA score	60 ± 5
Fasting plasma glucose (mmol/l)	5.8 ± 0.6
Fasting plasma insulin (pmol/l)	80 ± 52
2 h plasma glucose (mmol/l)	7.5 ± 2.8
HbA_1c_	
mmol/mol	38 ± 1
%	5.6 ± 1.5
Glucose clearance rate (ml kg^−1^ min^−1^)	325 ± 55

### Study design

**Fig. 1:**
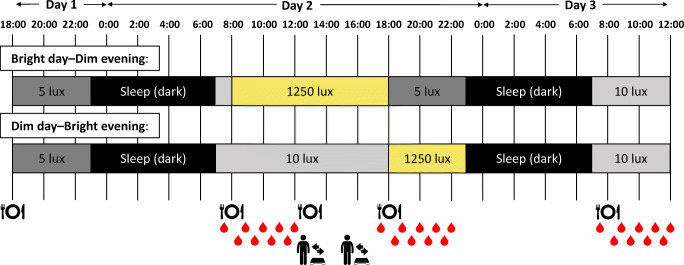
Study scheme. Fasted blood samples were taken at 07:45 h on days 2 and 3 and at 17:45 h on day 2. Postprandial blood samples were taken for 4 h at 30 min intervals after both breakfasts and dinner. Slow stepping exercise for 30 min was performed at 12:30 h preceding lunch and at 15:30 h

All volunteers underwent two 40 h sessions including two overnight stays in a respiration chamber (detailed study scheme, see Fig. 1). Each session started at 18:00 h on day 1 and ended at 12:00 h on day 3. In one session (Bright day–Dim evening), participants were exposed to bright light (1250 lx) during the daytime (08:00–18:00 h) and dim light (5 lx) during the evening (18:00–23:00 h). We chose 5 lx for the evening as generally the dim light melatonin onset (DLMO) is determined in dim light below 10 lx. In the other session (Dim day–Bright evening), participants were exposed to dim light (10 lx) during the daytime (08:00–18:00 h) and bright light (1250 lx) during the evening (18:00–23:00 h). We chose 10 lx for the daytime as 5 lx was considered too low and unpleasant to spend 10 h in. During the evening of the first day and the morning of the last day of both conditions, the light intensity was low at 5 and 10 lx, respectively. More detailed information on light characteristics is given in electronic supplementary material (ESM) (ESM Methods ‘Light characteristics’, ESM Table 1, ESM Fig. 1).

For 3 days before each session, participants were instructed to try to sleep every night from 23:00 to 07:00 h, to avoid caffeine and alcohol, to refrain from strenuous exercise and from exposure to excessive bright/dim light, and to follow normal eating patterns (three meals a day at ~08:00, 13:00 and 18:00). Participants filled in a food diary over each 3 day run-in period and food intake was kept similar during both sessions. The wash-out period between sessions was at least 4 days and not longer than 4 weeks.

### Actigraphy

For the entire 3 day pre-period and during their stay in the respiration chamber, participants wore an actigraph (Actiwatch Spectrum, Philips Respironics, Murrysville, PA, USA) on their wrist. Detected bedtime, wakeup time, immobile time during the sleep period (in %) and activity count were used as variables to: (1) monitor if participants adhered to the instructions (i.e. sleeping times) for the pre-period at home; and to (2) compare activity patterns and sleep characteristics between everyday life at home and under experimental conditions in the respiration chamber.

### Energy metabolism and study meals

Daily energy expenditure, sleeping metabolic rate (SMR) and substrate oxidation were calculated based on measured concentrations of oxygen consumption and carbon dioxide production (Omnical, Maastricht Instruments, the Netherlands) [13] according to Weir’s equations [14]. SMR (defined as lowest 2 h mean energy expenditure) during the first night was multiplied by an activity factor of 1.5 to calculate energy requirements. Breakfast (~25 energy percentage [E%]) was given at 08:00 h, lunch (30 E%) at 13:00 h, dinner (45 E%) at 18:00 h. Daily macronutrient composition was ~55 E% as carbohydrates, ~30 E% as fat (~9 E% saturated) and ~15 E% as protein. Breakfast and lunch were bread-based, and, thus, the percentage of energy from carbohydrates was higher compared with the dinner. Participants were instructed to finish each meal within 20 min while sitting at a table. No snacks or drinks other than water were provided between meals. The meals were equivalent in macronutrient and caloric content between the two experimental sessions, and it was ensured that participants ate all the provided food in both sessions. In the respiration chamber, the ambient temperature was set to 21°C and light exposure was strictly controlled according to the respective protocol. Participants were allowed to perform regular office work and were instructed to follow a daily activity programme (i.e. scheduled standing and stepping) to standardise activity between sessions and participants. The windows in the wall were shielded from the outside to ensure that no light could leak into the room.

### Skin and core body temperatures

According to ISO standard 9886 [15], skin temperatures were measured at 14 ISO-defined body sites using wireless temperature sensors (iButtons, Maxim Integrated Products, USA) [16]. Core body temperature (CBT) was measured using a telemetric pill (Vital Sense, Philips Healthcare, the Netherlands), which was ingested on the first evening after dinner. Participants wore a chest belt that receives and records the core pill’s signal (Equivital, Hidalgo, UK).

### Body composition

Body composition was determined on the last day of the intervention after leaving the respiration chamber at 12:30 h. Body mass and body volume were assessed using air-displacement plethysmography (BodPod, Cosmed, Italy) according to the manufacturer’s protocol, as previously reported [17].

### Blood sampling and analysis

Fasted blood samples were taken via an intravenous cannula placed in the forearm at 07:45 h on days 2 and 3 and at 17:45 h on day 2. Postprandial blood samples were taken every 30 min for 4 h after both breakfasts and the second dinner (see Fig. 1). Serum TG (Sigma, St Louis, USA) and plasma glucose (Horiba, Montpellier, France) levels were measured colorimetrically using a Cobas Pentra C400 analyser (Horiba, Montpellier, France). Insulin levels were measured in serum with an ELISA kit (Crystal Chem, Elk Grove Village, USA). Melatonin concentration was measured by LC-MS/MS. For detailed information and validation of the method, see van Faassen et al [18]. Intra-assay CV was 8.9% for low concentrations and 3.5% for high concentrations. Inter-assay CV was 9.5% for low concentrations and 4.2% for high concentrations. The lower limit of quantification was 1.9 pg/ml (8.18 pmol/l). The DLMO threshold was set to >10 pg/ml (43.05 pmol/l).

### Statistical analysis

Postprandial data were analysed using a generalised linear mixed model with time and light conditions and their interaction as fixed effects. This model takes missing values into account, which occurred due to the temporary non-functioning of the inserted cannula in some individuals (<5 individual missing data points for each blood marker out of up to 196 individual data points). Paired *t* tests were conducted to detect differences between conditions in most outcome measures without repeated measures. The level of significance was set at α <0.05 for all analyses. Data are presented as mean ± SEM if not stated otherwise. All statistical analyses were performed with the GraphPad Prism 8 software package (GraphPad Software, San Diego, USA).

## Results

### High compliance of participants to instructions before and during the intervention

The mean times participants went to bed and woke up during the 3 day pre-period at home and the 2 intervention days in the respiration chambers were determined based on actigraphy data (ESM Table 2). Activity patterns are shown in ESM Fig. 2. During their stays in the respiration chambers, participants were asked to go to bed at 23:00 h and wake up at 07:00 h with lights being switched off and on, respectively. Actigraphy data indicated that participants were lying quietly in bed a few minutes later and were active in the morning already a few minutes earlier. Detected bed and wakeup times did not differ over the 3 day pre-period and the intervention period between conditions. Also, no differences in physical activity were detected between conditions (ESM Fig. 2).

### Distal skin temperature is modulated by light exposure

**Fig. 2:**
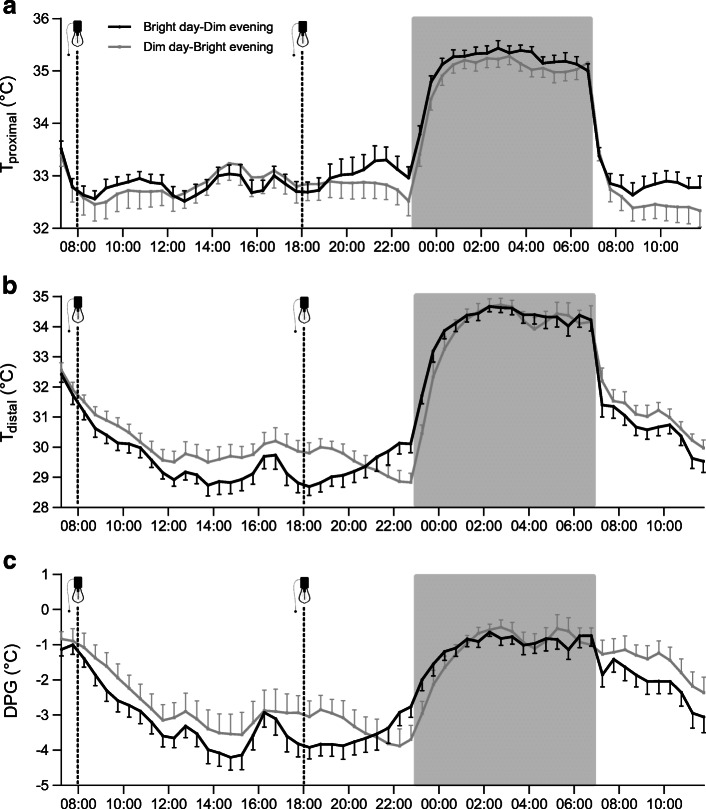
Overview of different skin temperature outcomes averaged over 30 min intervals (*n* = 13). T_proximal_ (**a**), T_distal_ (**b**) and DPG (**c**). The dashed lines with the light bulb indicate the time points when the light settings were changed. Data are presented as mean ± SEM

Proximal skin temperature (T_proximal_, mean of all measured skin regions of the torso) showed a clear day–night difference throughout the intervention due to the warming effect of the bed sheets covering the skin at night, but was not different between conditions (Fig. 2a). In both conditions, distal skin temperature (T_distal_, mean of foot and hand temperatures) decreased after waking up, reaching a plateau at ~12:00 h, which was interrupted by transient increases due to stepping exercise at 12:30 and 15:30 h (Fig. 2b). T_distal_ was lower at 18:00 h when spending the day (8:00–18:00 h) in bright light, compared with spending the day in dim light (28.8 ± 0.3°C vs 29.9 ± 0.4°C, *p*=0.039). After switching the light conditions at 18:00 h, T_distal_ increased and was eventually higher at 23:00 h in the Bright day–Dim evening condition compared with the Dim day–Bright evening condition (30.1 ± 0.3°C vs 28.8 ± 0.3°C, *p*=0.006). While T_distal_ increased over the evening (18:00–23:00 h) in the Bright day–Dim evening condition, it concurrently decreased in the Dim day–Bright evening condition (% change: +4.7 ± 1.3% vs −3.4 ± 0.5%, *p*<0.001). As T_proximal_ was not different between conditions, the distal–proximal skin temperature gradient (DPG) followed the same pattern as T_distal_ (Fig. 2c). The DPG, which can be used as an estimate of the degree of vasodilation [19], illustrates a delayed onset of vasodilation in the Dim day–Bright evening condition.

Due to technical issues with transmitting the CBT data from the telemetric pill, we obtained complete CBT datasets from only eight volunteers (ESM Fig. 3). The nadir of CBT was not phase shifted either from the first to the second night within any of the two light conditions (Bright day–Dim evening: 0:30 ± 17 min vs 0:32 ± 31 min, *p*=0.721; Dim day–Bright evening: 0:54 ± 117 min and 1:10 ± 88 min, *p*=0.771) or between light conditions in the second night (*p*=0.771). No further effect of the light intervention on CBT was detected (condition × time interaction: *p*=0.999).

### Plasma melatonin is strongly suppressed by bright evening light

**Fig. 3:**
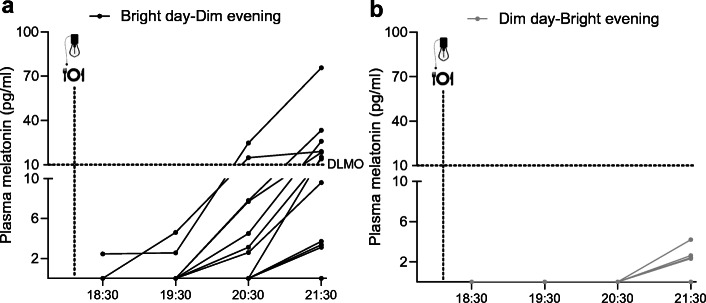
Plasma melatonin (*n* = 14) in the evening of day 2 upon Bright day–Dim evening (**a**) and Dim day–Bright evening (**b**). Lines represent individual data. Data points below the detection threshold of 1.9 pg/ml (8.18 pmol/l) are illustrated as 0 values. The horizontal dashed line indicates the DLMO threshold of 10 pg/ml (43.05 pmol/l). To convert melatonin values from pg/ml to pmol/l, please multiply by 4.305

In the Dim day–Bright evening condition, plasma melatonin was significantly suppressed compared with Bright day–Dim evening (condition × time interaction: *p*<0.001, condition: *p*=0.012; Fig. 3). Melatonin levels had exceeded the DLMO criterion of >10 pg/ml (43.05 pmol/l) in seven participants at 21:30 h upon Bright day–Dim evening (20.1 ± 6.3 pg/ml [86.14 ± 27.12 pmol/l], *n* = 11; Fig. 3a). Only two participants exceeded DLMO levels already at 20:30 h and the remaining five at 21:30 h. In contrast, upon Dim day–Bright evening, melatonin concentrations that exceeded the detection threshold of 1.9 pg/ml (8.18 pmol/l) could be detected in only four participants (Fig. 3b).

### Evening light exposure modulates postprandial glucose

**Fig. 4:**
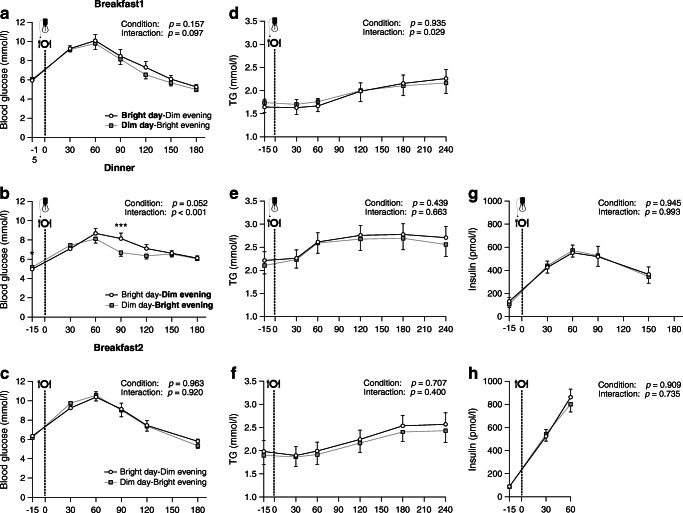
Postprandial plasma responses for the two meals on day 2 (Breakfast1 [*n* = 13] and Dinner [*n* = 14]) and breakfast on day 3 (Breakfast2 [*n* = 13]). Blood glucose (**a**) and TG (**d**) for the first breakfast; blood glucose (**b**), TG (**e**) and insulin (**g**) for the dinner; blood glucose (**c**), TG (**f**) and insulin (**h**) for the second breakfast. Postprandial data were analysed using a generalised linear mixed model with time and light conditions and their interaction as fixed effects. Data are presented as mean ± SEM; ***p<0.001, *p<0.05 (note that there is a single * symbol in Fig. 4b, partially obscured by the *y*-axis)

In the Bright day–Dim evening condition, postprandial plasma glucose after the first breakfast increased more compared with the Dim day–Bright evening condition, although this did not reach statistical significance (condition × time interaction: *p*=0.097; Fig. 4a). In addition, plasma TG after the first breakfast increased significantly more in the Bright day–Dim evening condition (condition × time interaction: *p*=0.029; Fig. 4b) as compared with the Dim day–Bright evening condition.

In the Bright day–Dim evening condition, plasma glucose was lower at 17:45 h, just before serving dinner and activating the evening light settings, compared with in the Dim day–Bright evening condition (5.0 ± 0.2 vs 5.2 ± 0.2 mmol/l, *p*=0.02). In the Bright day–Dim evening condition, glucose levels after dinner increased more compared with the Dim day–Bright evening condition (condition × time interaction: *p*<0.001; Fig. 4c), with a higher incremental AUC (394 ± 66 vs 307 ± 55, *p*=0.009). Plasma insulin and TG levels after dinner did not differ between conditions (condition × time interaction: *p*=0.993 and *p*=0.663; Fig. 4d,e).

On the last day of the intervention, after an overnight fast and under standardised dim light conditions, all plasma metabolites before and after breakfast were not differently affected by the prior differences in light exposure (Fig. 4f–h).

### The Dim day–Bright evening condition reduced the SMR and energy expenditure after dinner

**Fig. 5:**
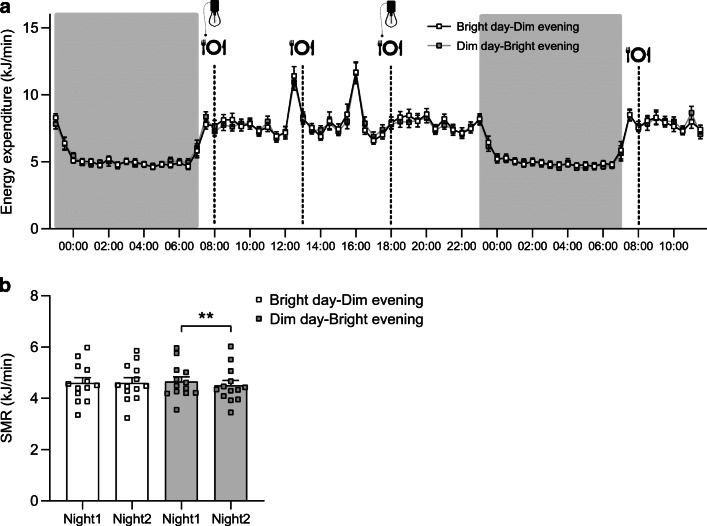
Energy expenditure over the entire time spent in the respiration chamber (**a**) and SMR of both nights per condition (*n* = 13; Night1 refers to the first baseline night spent in the respiration chamber without any differences in light intervention; Night2 refers to the second night after the respective light intervention) (**b**); *p* values are based on paired *t* tests; **p<0.01. Data are presented as mean ± SEM

The 24 h energy expenditure on day 2 (07:00 h on day 2 to 07:00 h on day 3, Bright day–Dim evening: 10,124 ± 394 kJ, Dim day–Bright evening: 10,036 ± 364 kJ; *p*=0.446) and the energy expenditure on day 3 (07:00–12:00 h, Bright day–Dim evening: 1884 ± 80 kJ, Dim day–Bright evening: 1909 ± 80 kJ; *p*=0.558) were not significantly different between light conditions (Fig. 5a). Furthermore, no differences between conditions could be detected in energy expenditure in the postprandial phase of the first breakfast (08:00–11:00 h: 1407 ± 63 kJ vs 1398 ± 54 kJ, *p*=0.751). However, in the postprandial phase after dinner, energy expenditure was higher in the Bright day–Dim evening condition (18:00–21:00 h: 1482 ± 63 kJ vs 1432 ± 63 kJ, *p*=0.044). The SMR was reduced in the night after the light intervention compared with the night preceding the light intervention, but only after Dim day–Bright evening (−3.3 ± 0.9%, condition × time interaction: *p*=0.013, *n* = 13; Fig. 5b). The SMR of the night after the light intervention was higher in the Bright day–Dim evening condition compared with the Dim day–Bright evening condition, although the difference was not significant (4.6 ± 0.2 vs 4.5 ± 0.2 kJ/min, *p*=0.065). The respiratory exchange ratio, calculated as the quotient of CO_2_ production and O_2_ consumption, was not different between conditions over any specific time interval (≥1 h) of the 40 h spent in the respiration chamber (ESM Fig. 4).

## Discussion

Optimising indoor light conditions to more closely mimic the natural light/dark cycle holds promise to prevent adverse effects on whole-body energy and glucose metabolism associated with the nowadays prevailing constant indoor lighting conditions. Here, we found that spending the day in bright light led to lower plasma glucose levels preceding the last meal of the day, whereas plasma glucose levels after that meal were higher after the Bright day–Dim evening condition, i.e. when the dinner was consumed under dim light conditions*.* The Bright day–Dim evening condition had a positive influence on energy expenditure: the SMR could be maintained compared with the night preceding the light intervention, whereas it dropped after the Dim day–Bright evening condition. Moreover, energy expenditure during dinner was increased in the Bright day–Dim evening condition. As expected, Bright day–Dim evening facilitated melatonin secretion in the evening, which was strongly suppressed in the Dim day–Bright evening condition. Furthermore, we found a lower T_distal_ at 18:00 h after spending the day in bright light and higher T_distal_ after spending the evening in dim light at 23:00 h (Bright day–Dim evening). Postprandial plasma TG levels were higher when breakfast was consumed under bright light conditions. Together, these findings suggest that the indoor light environment of insulin-resistant individuals can modulate metabolic variables in a time-of-day-dependent manner to influence metabolic health in the long term.

The role of melatonin as a mediator between environmental light and mammalian metabolism is well established. Melatonin secretion by the pineal gland requires darkness after sunset, with the inhibitory signal for light-induced melatonin suppression ultimately coming from intrinsically photosensitive retinal ganglion cells [20, 21]. The suppression of melatonin upon bright light during the evening in the present study is hence not surprising. Melatonin started to rise 2–4 h before habitual bedtime upon Bright day–Dim evening, which is in line with the reported range of DLMO in young healthy volunteers [22–24]. In contrast, CBT data (*n* = 8) consistently indicated a relatively early occurrence of the CBT nadir (range of the mean of all nights between 00:00 and 01:15 h) in line with another study in insulin-resistant men of similar age [25], which seems to be phase-advanced compared with younger participants (i.e. between 04:00 and 06:00 h [26, 27]). Also, chronotype assessment characterised our volunteers as being of moderate morning type on average (MEQ-SA: 60 ± 5) which is typical in the aged population [28].

The interplay between melatonin and human glucose metabolism has recently gained a lot of interest [29] due to evidence for adverse effects of circadian misalignment (e.g. night-shift work) on glucose metabolism [26, 30] and the discovery of type 2 diabetes mellitus risk variants in the melatonin receptor 1B gene, i.e. *MTNR1B* [31, 32]. *MTNR1B* has even been found to be a predictor for the transition from normoglycaemia to glucose intolerance [33]. Moreover, melatonin supplementation in the morning has been shown to acutely reduce glucose tolerance in aged women [34]. In the present study, we observed higher postprandial glucose levels after dinner in the Bright day–Dim evening condition compared with Dim day–Bright evening, with the greatest difference 90 min after the meal, at 19:30 h. As melatonin was strongly suppressed by bright light in the evening, one could assume that the suppression of melatonin was beneficial for glucose homeostasis. However, it should be noted that under dim light conditions, melatonin onset occurred for most volunteers at 20:30–21:30 h, whereas the light effects on postprandial glucose occurred earlier. Therefore, the effects of the light condition on postprandial glucose can probably not be explained by its effect on melatonin.

The reduced postprandial glucose levels after the dinner meal in the Dim day–Bright evening condition occurred also in the absence of differences in insulin levels. Sone et al [35] demonstrated that gastric motility and carbohydrate absorption (determined by breath hydrogen excretion) were both decreased in young women consuming an evening meal at 17:00 h after spending the day from 07:00 to 15:00 h in dim light (80 lx) compared with bright light (5000 lx). These effects were accompanied by higher parasympathetic activity in the evening after spending the day in bright light, which is likely to promote gut functioning and thereby absorption of carbohydrates [36]. In support, Tokura et al [37] found that secretion of saliva in response to food stimulation was influenced by the light environment in a time-of-day-dependent manner, with enhanced saliva secretion after bright light during the daytime and suppressed secretion after bright light in the evening. Enhanced secretion of saliva facilitates subsequent gastrointestinal digestion of complex carbohydrates through the presence of salivary amylase. Taken together, combining bright light during the daytime and dim light during the evening may promote digestion of food and could contribute to our findings of increased postprandial glucose levels after the dinner meal. Future research integrating appropriate methodology such as a meal tracer to quantify gastrointestinal processes is required to further support this claim. Albreiki et al [4] only investigated the acute metabolic effects of LAN in young healthy volunteers without taking prior light exposure during the daytime into account, and found, in contrast to our findings, negative effects of LAN on glucose tolerance. However, in that study, the meal challenge was also given later at 22:00 h. In line with Albreiki et al [4], Cheung et al [11] showed that both morning and evening blue-enriched light exposure (for 3 h during mealtime) increased postprandial insulin levels and HOMA-IR compared with a constant 24 h dim light condition in young healthy volunteers. In the present study, the abrupt switch of the light conditions at 18:00 h from bright to dim or vice versa, which precisely coincided with dinner time, could explain why our results contradict previous reports. Ultimately, how LAN influences glucose homeostasis may depend on prior light exposure before giving the meal challenge, how late into the evening food intake takes place, and the state of glucose tolerance and age of the studied population.

A time-of-day dependency of the effects of light exposure on glucose metabolism becomes evident when considering that we found the opposite pattern in the morning, with a tendency towards a greater increase in postprandial glucose upon bright light in the morning during/after breakfast, although this did not reach statistical significance. In comparison with Versteeg et al [9], who found significantly increased postprandial glucose levels in type 2 diabetes mellitus patients who were exposed to a brighter morning light stimulus (i.e. 4000 lx vs 1250 lx in our study), our data now suggest that either higher brightness levels are needed to modulate the postprandial glucose response after breakfast, or the modulatory effect depends on the severity of glucose intolerance of the studied population. In line with Versteeg et al [9], our data confirm that postprandial TG levels after breakfast are consistently increased upon bright light in the morning.

In accordance with earlier findings in young healthy individuals [38, 39], we found that the environmental light strongly modulates T_distal_ also in insulin-resistant volunteers. T_distal_ is lowered by vasoconstriction of the peripheral skin and can serve to increase CBT. Our data suggest that this effect was potentiated upon bright light during the daytime, whereas vasodilation to lower CBT, which promotes a rapid onset of sleep [19], was only facilitated upon dim light during the evening. Vasodilation upon dim light in the evening is mostly mediated through reduction of the sympathetic vasoconstrictor tonus [40]. Overall, Bright day–Dim evening induced a diurnal pattern in T_distal_ and the DPG. This supports a healthy diurnal variation in blood pressure with higher values during the daytime and lower values at night [41–43]. Therefore, the combination of bright light during the daytime and dim light during the evening seems promising to improve diurnal variation in blood pressure in a population that is prone to developing cardiovascular complications in the long term.

A novel finding of our study is that prior light exposure can influence the SMR. When comparing the night following the Dim day–Bright evening scheme compared with the previous night, SMR dropped by 3.3 ± 0.9%. Following the Bright day–Dim evening scheme, the SMR was maintained. Previous studies have found that resting energy expenditure (REE; i.e. assessed during wakefulness in contrast to SMR) shows circadian rhythmicity [44], and that circadian misalignment, such as sleep restriction and night-shift work, can alter energy expenditure [26, 45, 46]. Since light exposure acts as a zeitgeber to align endogenous physiological rhythms to the environmental light/dark cycle, it is tempting to speculate that light exposure may misalign the diurnal variation in REE. More research is needed to investigate how light exposure could affect energy expenditure, especially since small changes in energy metabolism can contribute to the development of obesity and metabolic diseases.

In conclusion, by performing detailed 24 h metabolic phenotyping by means of frequent blood sampling, continuous indirect calorimetry and skin temperature assessment, we demonstrate that the timing of light exposure can influence postprandial substrate handling, energy expenditure and thermoregulation of insulin-resistant individuals. In contrast to the Dim day–Bright evening condition, the Bright day–Dim evening condition mostly elicited favourable outcomes: lower plasma glucose levels preceding the dinner meal, greater energy expenditure in response to the dinner meal, conservation of SMR and facilitation of a diurnal pattern in T_distal_. The finding that postprandial TG levels after breakfast and postprandial glucose levels after dinner were less increased in the Dim day–Bright evening condition warrants further investigation. In the future, more research is required to exploit different light regimens in office buildings and home settings for their potential to prevent metabolic diseases.

## Supplementary Information

ESM (PDF 1467 kb)

## Data Availability

The datasets generated during and/or analysed during the current study are available from the corresponding author on reasonable request.

## Abbreviations

CBT: Core body temperature
DLMO: Dim light melatonin onset
DPG: Distal–proximal skin temperature gradient
E%: Energy percentage
LAN: Light at night
MEQ-SA: Morningness–Eveningness Questionnaire Self-Assessment
REE: Resting energy expenditure
SMR: Sleeping metabolic rate
T_distal_: Distal skin temperature
TG: Triacylglycerol
T_proximal_: Proximal skin temperature
