# Supplementary material for: The influence of bright and dim light on substrate metabolism, energy expenditure and thermoregulation in insulin-resistant individuals depends on time of day
Source: Diabetologia. 2022 Feb 2;65(4):721–32. doi: 10.1007/s00125-021-05643-9 (PMC8894310; doi:10.1007/s00125-021-05643-9)
Supplement: Supplementary file 1 — (PDF 1467 kb) [file 125_2021_5643_MOESM1_ESM.pdf]

Electronic supplementary material (ESM):

ESM Methods:

Light characteristics

In the respiration chamber, the installed light system consisted of light-emitting diode (LED) wall washers (Philips SkyRibbon IntelliHue Wall Washing Powercore). By combining and precisely controlling multiple channels of the LED light, the system can produce white light. All applied light conditions were set to a correlated colour temperature of 4000 K while aiming for minimal differences in spectral composition. The spectrum of each lighting condition (5, 10, 1250 lux) was measured once using a radiospectrometer (Lighting passport, Asensetek). Measurements were carried out vertically in the outward direction of the optical axis at the eye level simulating once a subject facing the computer when sitting at the desk and once facing the TV sitting in bed (see ESM Figure 1 for positions of measuring points). These two positions were selected as volunteers usually spent most of their time in the respiration chamber in this manner of directing their view. The collected data was extracted using the software Spectrum Genius (Asensetek) and further converted from relative to absolute irradiance values (by https://luox.app [2]). Illuminance, -opic (ir)radiances [3] and spectral irradiances of all conditions are reported according to Spitschan et al. [4] in ESM Table 1.

ESM Table 1. Light stimulus specification.

Location of measurement	Bed			Desk		
Condition	DL_5	DL_{10}	BL_{1250}	DL_5	DL_{10}	BL_{1250}
Illuminance [lux]	3.40	7.90	1397.20	2.60	6.00	1161.70
S-cone-opic irradiance (mW/m^2)	1.41	2.97	562.97	1.16	2.30	447.63
M-cone-opic irradiance (mW/m^2)	4.42	10.15	1811.53	3.40	7.72	1497.20
L-cone-opic irradiance (mW/m^2)	5.55	12.89	2276.76	4.24	9.79	1894.43
Rhodopic irradiance (mW/m^2)	3.81	8.51	1536.52	2.98	6.50	1256.95
Melanopic irradiance (mW/m^2)	3.20	7.05	1287.88	2.52	5.40	1049.09
S-cone-opic EDI [lux]	1.72	3.64	688.91	1.42	2.82	547.73
M-cone-opic EDI [lux]	3.03	6.98	1244.45	2.34	5.30	1028.53
L-cone-opic EDI [lux]	3.41	7.91	1397.71	2.61	6.01	1163.09
Rhodopic EDI [lux]	2.63	5.87	1060.07	2.05	4.48	867.16
Melanopic EDI [lux]	2.41	5.32	971.28	1.90	4.07	791.16

Open https://doi.org/10.5287/bodleian:AeZAXN1km (light conditions in bed) or https://doi.org/10.5287/bodleian:dp7QEqYAm (light conditions at desk) and follow "Related items" to access the spectral irradiance values (in $W/(m^2 \cdot nm)$) of the entire wavelength spectrum of the light stimulus at the respective measurement location.

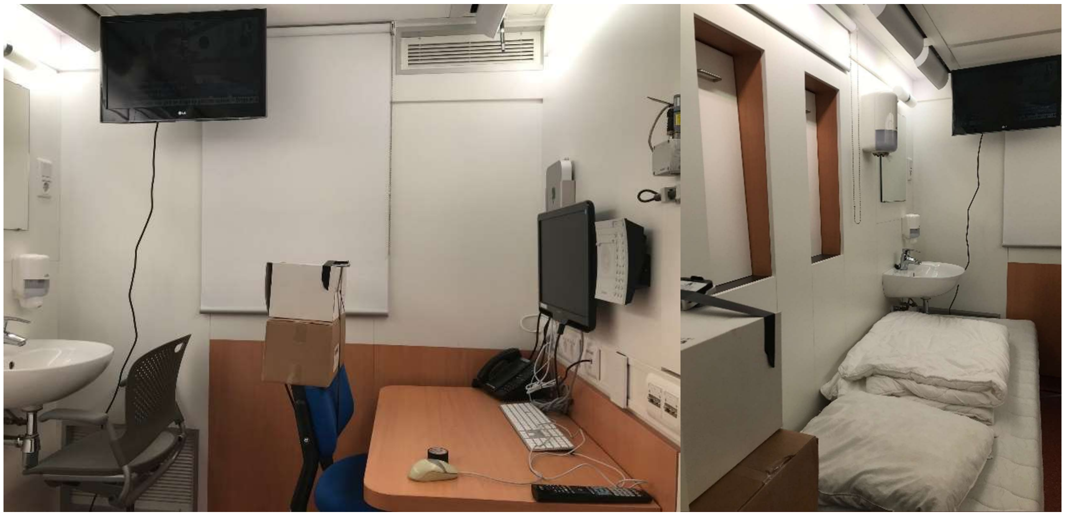

ESM Figure 1. Spectrometer measurement set-ups. The left panel shows the measurement conducted at the eye level of the subject facing the computer when sitting at the desk. The right panel shows the measurement conducted at the eye level of the subject facing the TV sitting in bed. For both measurements, the TV and computer were switched on with their brightness levels adjusted according to the dim (5 or 10 lux) or bright (1250 lux) light condition.

ESM Results:

ESM Table 2. Sleep characteristics of the participants over the 3-day pre-period at home and the 2-day intervention period spent in the respiration chamber (measured by actigraphy). Individual bedtimes in the home-setting ranged from 21:00 to 1:00.

	3-day pre-period					
	Day 1		Day 2		Day 3	
	Bedtime	Wakeup time	Bedtime	Wakeup time	Bedtime	Wakeup time
BD-DD	23:26 ± 26	7:05 ± 31	23:31 ± 44	7:12 ± 36	23:30 ± 33	7:00 ± 31
DD-BD	23:31 ± 32	6:49 ± 30	23:26 ± 41	7:01 ± 23	23:18 ± 23	6:57 ± 27

	2-day intervention period			
	Day 1		Day 2	
	Bedtime	Wakeup time	Bedtime	Wakeup time
BD-DD	23:05 ± 6	6:55 ± 11	23:08 ± 11	6:51 ± 17
DD-BD	23:12 ± 18	6:50 ± 22	23:12 ± 19	6:47 ± 27

Data are shown as mean ± standard deviation (*n*=13); BD-DD: Bright day–Dim evening; DD-BD: Dim day–Bright evening; p-value based on paired t-tests

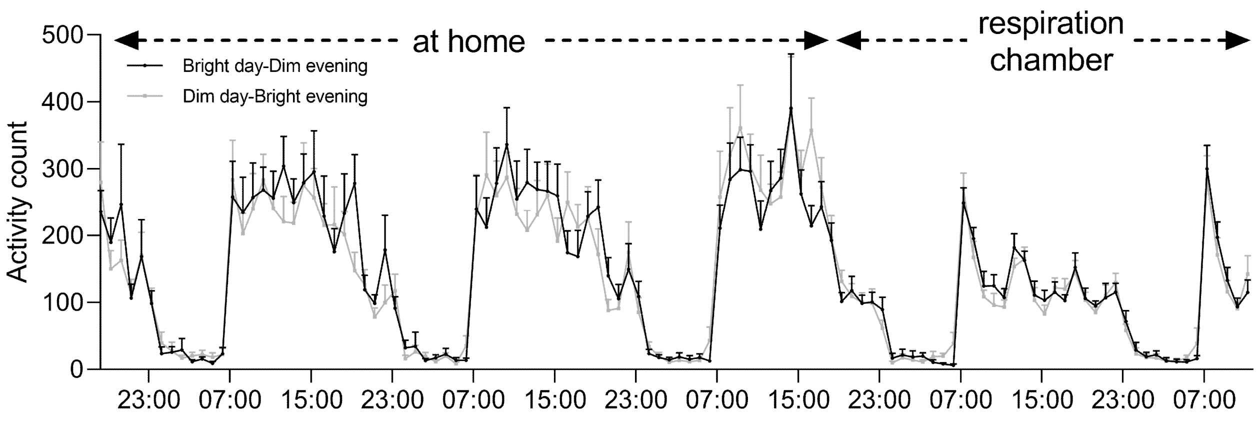

ESM Figure 2. Activity count (measured by the actigraph, *n*=13) over the 3-day pre-period and 2-day intervention period. No significant differences were observed over time between the two different light conditions. Data are averaged over 30 min intervals and presented as mean ± SEM.

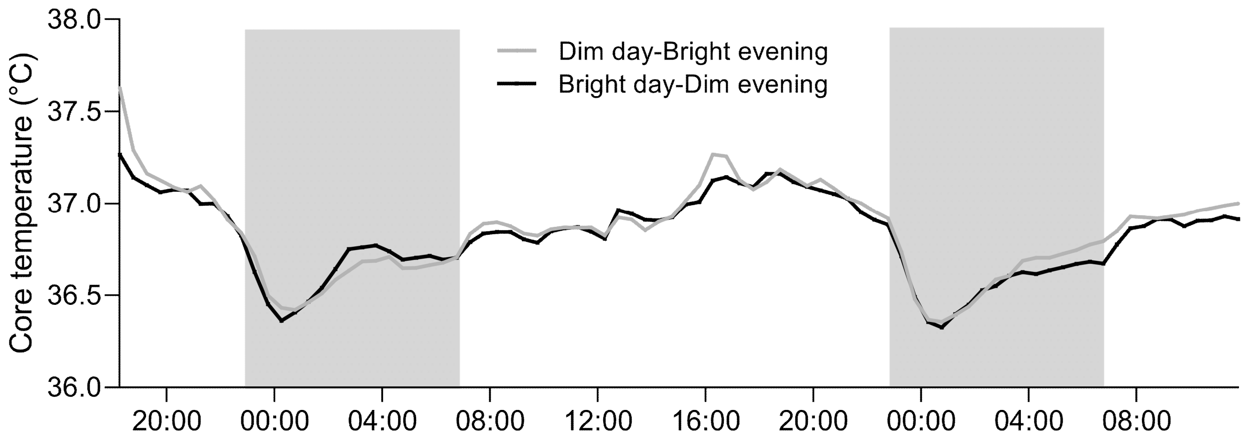

ESM Figure 3. Core body temperature (CBT) averaged over 30 min intervals (*n*=8).

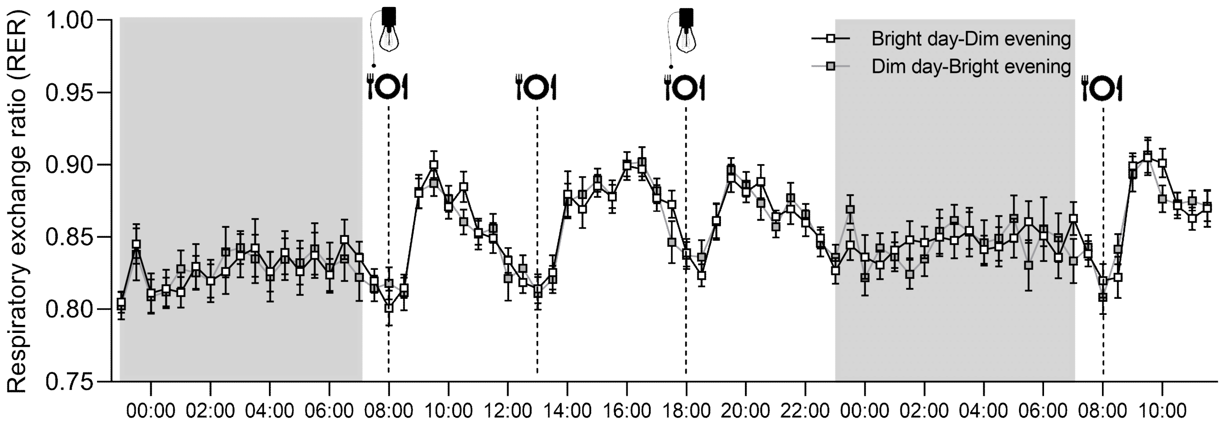

ESM Figure 4. Respiratory exchange ratio shown over the entire time spent in the respiration chamber. Open squares indicate *Bright day–Dim evening* and grey squares indicate *Dim day–Bright evening*. Data are averaged over 30 min intervals and presented as mean ± SEM (*n*=14).

ESM References:

[1] Plasqui G, Soenen S, Westerterp-Plantenga MS, Westerterp KR (2011) Measurement of longitudinal changes in body composition during weight loss and maintenance in overweight and obese subjects using air-displacement plethysmography in comparison with the deuterium dilution technique. Int J Obes 35(8):1124–1130

[2] Spitschan M, Mead J, Roos C, et al (2021) luox: novel validated open-access and open-source web platform for calculating and sharing physiologically relevant quantities for light and lighting. Wellcome Open Res 6:69

[3] CIE S 026/E:2018 CIE System for Metrology of Optical Radiation for IpRGC-Influenced Responses to Light. International Commission on Illumination; 2018. doi:10.25039/s026.2018

[4] Spitschan M, Stefani O, Blattner P, Gronfier C, Lockley SW, Lucas RJ (2019). How to Report Light Exposure in Human Chronobiology and Sleep Research Experiments. Clocks Sleep. 1(3):280-289.
